# Demand for Weight Loss Counseling After Copayment Elimination

**DOI:** 10.5888/pcd10.120163

**Published:** 2013-04-04

**Authors:** Matthew L. Maciejewski, William S. Yancy, Maren Olsen, Hollis J. Weidenbacher, David Abbott, Morris Weinberger, Santanu Datta, Leila C. Kahwati

**Affiliations:** Author Affiliations: William S. Yancy Jr, Durham Veterans Affairs and Duke University Medical Center, Durham, North Carolina; Maren Olsen, Durham Veterans Affairs and Duke University, Durham, North Carolina; Hollis J. Weidenbacher, David Abbott, Durham Veterans Affairs, Durham, North Carolina; Morris Weinberger, Durham Veterans Affairs, Durham, North Carolina, and University of North Carolina, Chapel Hill, North Carolina; Santanu Datta, Duke University Medical Center, Durham, North Carolina; and Leila C. Kahwati, Department of Veterans Affairs, Durham, North Carolina.

## Abstract

**Introduction:**

Overweight and obesity are public health issues in the United States, and veterans have a higher rate of overweight and obesity than the general population. Our objective was to examine whether copayment elimination increased use of a weight loss clinic by veterans.

**Methods:**

We examined clinic use by 44,411 new patients seen in a Veterans Affairs (VA) MOVE! weight management clinic before the copayment elimination and clinic use by 64,398 new patients seen in the year after copayment elimination. We examined clinic use via mixed-effects models for patients who were already exempt from copayment and patients who were newly exempt from copayment. We used 2 outcomes before and after copayment elimination: 1) the ratio of number of clinic visits by new users with the mean number of MOVE! clinic visits by all users, and 2) the number of clinic visits by each new user in the 6 months after their first visit. All models were adjusted for patient and clinic factors.

**Results:**

Among newly exempt patients, the clinic-standardized rate of new use increased by 2.2% after the copayment was eliminated but increased 12% among already exempt veterans. This finding was confirmed in adjusted analyses. Analysis of number of clinic visits adjusted for patient and clinic factors also found that exempt and nonexempt veterans had similar numbers of repeat clinic visits.

**Conclusion:**

We saw an unexpected larger increase in demand among veterans who receive all VA care for free. These results suggest that VA should not assume that copayment reductions for selective preventive services will motivate patient change and achieve intended system-level outcomes.

## Introduction

Obesity is a major health and health care problem; more than one-third of Americans are currently obese and another approximately one-third are overweight (1). Obesity is associated with chronic, debilitating illnesses (2) and was the second leading cause of preventable deaths in the United States in 2005 (3). Excess weight is also costly, resulting in an estimated $75 to $93 billion in annual medical care expenditures (4). Veterans have a higher rate of overweight and obesity than the general population (5). Moreover, obesity rates were higher among veterans who obtained care partially (28%) or exclusively (31%) in Veterans Affairs Medical Centers (VAMCs) than veterans who did not use VA services (24%).

Despite widespread dissemination of evidence-based weight management recommendations, only 20% of people trying to lose weight adhere to guidelines on dietary intake and physical activity (6). Patients who successfully lose weight typically regain much of that weight after 1 year and nearly all of it by 5 years (7). Therefore, the US Preventive Services Task Force has recommended only high-intensity counseling interventions (at least 2 person-to-person sessions per month for at least the first 3 months of an intervention) (8).

To address the increasing prevalence of overweight and obesity in veterans, the Veterans Health Administration (VHA) developed the MOVE! Weight Management Program for veterans in 2006. The program provides a systematic, evidence-based approach to weight management for the nearly 6 million veterans served by approximately 153 hospitals and 956 outpatient clinics (9). MOVE! uses a pretreatment intake questionnaire (http://www.move.va.gov/move23.asp) to develop tailored, behaviorally based dietary changes and physical activity self-management support that are delivered through individual or group face-to-face counseling and that are supported with interventions that use weight loss medications, intensive outpatient programs, and bariatric surgery. More than 40% of veterans who participated in at least 2 MOVE! treatment sessions lost at least 5% of their body weight in the short term; only 12.5% of nonparticipants matched for age, sex, comorbidities, and body mass index lost this amount (10).

In 2006, a $15 copayment was required by veterans for all face-to-face MOVE!-related clinic visits if they did not meet disability or income thresholds for copayment exemption. Veterans were exempt from copayments for MOVE! clinic visits if they had sufficient military-service–related disability or had a low income (“always exempt”). On June 15, 2008, VHA policy makers eliminated the copayment because patients and clinic staff expressed concern that the copayment was a barrier to initial and ongoing participation. The copayment change was communicated to VA staff at local sites but not to veterans through a coordinated national communications strategy. The policy to eliminate copayments is consistent with value-based insurance design principles that link cost sharing directly to clinical value so that cost-effective or cost-saving products and services have lower copayments than less cost-effective products and services (11). Value-based insurance design may become more prevalent because it was mandated per section 2713 of the Patient Protection and Affordable Care Act of 2010 (12). Studies have examined the effect of copayment elimination on medication use, but none have examined the effect of copayment elimination on weight loss service use.

The objective of this study was to evaluate the effect of the MOVE! copayment elimination on use of the MOVE! clinic by patients who had not previously received MOVE! clinic services. We examined whether the ratio of first-time MOVE! clinic visits to mean overall clinic use (ie, the clinic-standardized rate of new use) increased after the copayment was eliminated. We also examined whether new users increased the number of visits they made to the MOVE! clinic in the 6 months after their first visit.

## Methods

### Design

The Durham VAMC Human Subjects Protection committee reviewed and approved this study. We employed a cross-sectional pre–post study design with a nonequivalent control group. We had 2 distinct and nonoverlapping cross-sectional cohorts: 1) a preperiod (June 16, 2007, through June 15, 2008) cohort of veterans who were new MOVE! clinic users in the year before the copayment elimination and 2) a postperiod (June 16, 2008, through June 15, 2009) cohort of veterans who were new MOVE! clinic users in the year after the copayment elimination. No patients in the preperiod cohort were represented in the postperiod cohort. Patients were identified as new users if they had no MOVE! clinic use in the 12 months before the index visit (their first MOVE! clinic visit) in the preperiod or postperiod. The cohort included incident, rather than prevalent, MOVE! users to enable us to identify immediate effects (13) of the copayment elimination among a subgroup of MOVE! users who may have been more responsive to the policy change.

Although nonexempt veterans during the preperiod were required to pay a $15 copayment until the copayment was eliminated on June 15, 2008, for the purpose of this study we refer to veterans in the preperiod (and the postperiod) who did not meet requirements for exemption and for whom the MOVE! copayment was eliminated as the “newly exempt” cohort. We hypothesized that copayment elimination would most affect the behavior of new clinic users in the postperiod. We compared MOVE! clinic visits by newly exempt veterans to clinic visits by a subgroup of veterans in the preperiod and postperiod cohorts who were exempt from copayments for all outpatient clinic visits. Seventeen percent of the preperiod cohort were veterans who were required to pay copayments, and 16% of the postperiod cohort were newly exempt veterans who became newly exempt in the year after copayment elimination.

### Sample

The sample included 126 VAMCs with high-volume MOVE! clinics, defined as having 30 or more MOVE! participants during the preperiod. This threshold was chosen to identify VAMCs that had moved beyond the start-up phase of MOVE! implementation. For the clinic-standardized rate of new MOVE! use, we aggregated data to the clinic level by month for new users in the preperiod and the postperiod. The number of MOVE! visits by new users in the 6 months after their index visit was defined for 21,193 preperiod and 26,520 postperiod new users. We excluded 23,218 preperiod new users from the analysis of attendance precopayment and postcopayment removal because their first MOVE! clinic visit occurred within 6 months of the copayment elimination (between December 16, 2007, and June 14, 2008), so they had less than 6 months of preperiod time to make a repeat visit. We excluded 37,878 postperiod new users whose first visit occurred between December 16, 2008, and June 14, 2009, for parallel cohort construction. The final sample included 44,411 preperiod and 64,398 postperiod new users.

### Data sources and measures

To construct the 2 MOVE! clinic use outcomes and most patient characteristics (age, sex, race, copayment exemption status, and marital status), we used 2006–2009 VHA Outpatient Care File data, which contains information on every VHA outpatient visit. The first clinic use outcome was measured monthly at the clinic level separately for exempt and newly exempt users (126 clinics × [24 months × 2 copay groups] = 6,048) during the preperiod. This outcome was a ratio in which the numerator was the number of MOVE! first-time visits within each clinic by patients who were new users, and the clinic-specific denominator was the overall mean monthly number of MOVE! visits to that clinic. This ratio, which we refer to as the clinic-standardized rate of new MOVE! use, represents the ratio of first-time MOVE! clinic visits to mean overall clinic use by all users (prior users and new users). The denominator was constant during the 24 months and the 2 copay groups, ranging from 0.0 to 4.3, so the ratio varied only according to the monthly change in the number of veterans who were using the MOVE! clinic for the first time. The denominator reflected the overall mean number of MOVE! clinic visits, so it was possible for the number of MOVE! clinic visits by new users in a given month to be larger than the overall monthly mean preperiod use (eg, the ratio was greater than 1.0). The second use outcome was a count variable that measured at the patient level in both the preperiod and postperiod (n = 47,713), and consisted of the number of MOVE! visits by new users in the 6 months after their first clinic visit.

To control for comorbidity, we obtained Diagnostic Cost Group (DCG) risk-adjustment scores for each patient in the preperiod and postperiod cohorts. DCG scores have been as predictive of veterans’ 1-year expenditures and mortality as other comorbidity scores (14,15). Patient characteristics were aggregated to the clinic level for assessing new users because the outcomes and unit of analysis were clinic-year. Finally, we obtained details on MOVE! clinic characteristics from VHA MOVE! annual reports. This report, required by VHA, completed by each facility’s MOVE! coordinator, and reviewed by facility leadership before submission, measures program implementation and MOVE! staffing levels at each VAMC. We selected clinic-level characteristics that may affect a clinic’s ability to meet new demand that resulted from elimination of the MOVE! copayment, including having a local MOVE! coordinator, the coordinating committee’s make-up and linkage to primary care, and the availability of facility space for meetings and exercise sessions.

### Analysis

The analysis for the first outcome was a linear mixed-effects model based on the framework of a split-plot design (16). In this design, the clinic was the “whole plot,” and the 2 factors (treatments) of interest were exempt versus nonexempt and preperiod versus postperiod (ie, copayment elimination phase), where exempt versus nonexempt is the “whole plot factor” and preperiod versus postperiod is the “subplot factor.” Therefore, the linear mixed-effects model included fixed effects for copayment exemption status, copayment elimination phase (ie, preperiod or postperiod), and their interaction; and random effects for clinic and the clinic by copayment exemption status interaction. We included a third random effect for month, nested in the copayment exemption status by copayment elimination phase interaction. This model also controlled for clinic average age, average DCG score, proportion female, proportion white, and proportion married as additional fixed effects in the model.

We used a negative binomial mixed-effects model to estimate differences in the number of MOVE! clinic visits. The fixed effects in the model included the main factors of interest: an indicator for copayment eligibility, an indicator for the preperiod or postperiod cohort, and an interaction of the copay eligibility and pre–post indicators. This interaction term indicates whether use of the MOVE! clinic changed significantly in exempt vs nonexempt veterans between the preperiod and postperiod. Additional fixed effects included veterans’ age, DCG score, sex, race, and marital status. A clinic-level random effect was included in the model to account for the intraclass correlation of patients from the same clinic. All analyses were conducted using SAS version 9.2 (SAS Institute, Inc, Cary, North Carolina) and Stata version 10 (StataCorp LP, College Station, Texas).

## Results

### Ratio of first-time MOVE! clinic visits to mean overall clinic use by all users

During the year before the copayment elimination, the mean clinic-standardized rate of new MOVE! clinic use was higher for new users who were copayment exempt (18.0%) than for those required to pay copayments (3.6%), which suggests that for every 2 nonexempt veterans newly using MOVE! in the preperiod, 10 exempt veterans newly used MOVE! The mean clinic-standardized rate by veterans exempt from copayments and new to the MOVE! program increased from 18% to 30% of all clinic visits, while the mean clinic-standardized rate by veterans formerly required to pay copayments and new to the MOVE! program increased from 3.6% to 5.8%. Compared with nonexempt new users, the relative increase in the clinic-standardized rate new MOVE! clinic visits by exempt veterans was 12% in the year before the copayment elimination and 2.2% after. In the adjusted analysis (Table 1), we found a significant interaction between copayment elimination phase and copayment exemption status (−0.10, 95% confidence interval [CI], −0.15 to −0.04).

**Table 1:** Adjusted Model Estimates (95% CI) of Clinic-Standardized Rate of New MOVE! Clinic Use^a^^,^ b

Variable	Estimate (95% CI)	*P* Value
Newly exempt	−0.24 (−0.29 to −0.20)	<.001
Postperiod	0.03 (−0.02 to 0.06)	.22
Newly exempt × postperiod interaction	−0.10 (−0.15 to −0.04)	<.001
Average age of clinic patients	0.02 (0.01 to 0.02)	<.001
Proportion female	−0.004 (−0.006 to −0.002)	<.001
Proportion married	−0.0004 (−0.002 to 0.001)	.54
Proportion white	0.001(−0.001 to 0.001)	.25
Average comorbidity risk via diagnostic cost group score	−0.38 (−0.48 to −0.28)	<.001
MOVE! clinic coordinated by primary care	−0.07 (−0.12 to −0.01)	.01
MOVE! coordinated by a dietitian	−0.02 (−0.07 to 0.02)	.36
MOVE! coordinating committee includes . . .		
Nonprimary care nurse	0.02 (−0.03 to 0.07)	.45
Primary care physician	0.003 (−0.04 to 0.05)	.91
Midlevel provider	0.04 (−0.01 to 0.09)	.09
Health educator	−0.01 (−0.06 to 0.04)	.68
MOVE! clinic has sufficient space to meet needs	0.04 (−0.01 to 0.08)	.09
MOVE! indoor exercise facility is sufficient to meet needs	−0.03 (−0.09 to 0.03)	.31
MOVE! outdoor exercise facility is sufficient to meet needs	−0.03 (−0.08 to 0.02)	.19
Distinct weight maintenance groups	−0.05 (−0.09 to −0.004)	.03
Sample size	6,048	

Abbreviation: CI, confidence interval.

a Estimates, 95% CIs and *P* values derived from linear mixed-effects model. Preperiod (June 16, 2007, through June 15, 2008) is the cohort of veterans who were new MOVE! clinic users in the year before copayment elimination and postperiod (June 16, 2008, through June 15, 2009) is the cohort of veterans who were new MOVE! clinic users in the year after the copayment elimination.

b The clinic-standardized rate of new MOVE! clinic use is defined as ratio in which the numerator was the number of MOVE! first-time visits within each clinic by patients who were new users, and the denominator was the overall mean monthly number of MOVE! visits to that clinic in the preperiod.

### Number of MOVE! clinic visits by new users

In the preperiod and postperiod, compared with nonexempt patients, exempt patients were younger (mean age, 55 y vs 63 y), more likely to be female (12% vs 7%), less likely to be white (50% vs 54%), and less likely to be married (52% vs 70%) and had higher mean DCG scores (0.75 vs 0.61) (all *P* < .001, Table 2). The unadjusted mean number of MOVE! clinic visits in the 6 months after the first visit was 3.64 (standard deviation [SD] = 4.55) by exempt veterans and 3.63 (SD = 4.22) by nonexempt veterans in the preperiod and 3.59 (SD = 4.82) and 3.60 (SD = 4.97) in the postperiod, which corresponds to $54 per person ($15/visit × 3.6 visits) in waived copayments.

**Table 2:** Characteristics of Preperiod and Postperiod Cohorts of MOVE! Clinic Users^a^

Characteristic	Preperiod		*P* Value	Postperiod		*P* Value
	Copay Exempt	Newly Exempt		Copay Exempt	Newly Exempt	
Age, mean (SD), y^b^	55.3 (11.5)	62.7 (10.6)	<.001	54.7 (11.6)	61.2 (10.8)	<.001
Female, %	12	7	<.001	12	8	<.001
White race, %	50	54	<.001	51	57	<.001
Married, %	52	70	<.001	49	65	<.001
Mean Diagnostic Cost Group score^b^	0.75 (0.71)	0.61 (0.50)	<.001	0.71 (0.68)	0.55 (0.45)	<.001
Sample size	17,504	3,689	NA	22,145	4,375	NA

Abbreviation: NA, not applicable.

a Preperiod (June 16, 2007, through June 15, 2008) is the cohort of veterans who were new MOVE! clinic users in the year before copayment elimination and postperiod (June 16, 2008, through June 15, 2009) is the cohort of veterans who were new MOVE! clinic users in the year after the copayment elimination.

b Differences in age and Diagnostic Cost Group scores estimated via 2-sided *t* tests and differences in sex, race, and marital status estimated via χ^2^ tests.

On the basis of the interaction between newly exempt and postperiod indicators in the adjusted analysis, we found no change from the preperiod to postperiod in the number of visits by new users who were exempt versus those who were required to pay copayments (Table 3). Veterans in the postperiod had a lower visit rate (0.98; 95% CI, 0.96-0.99) to MOVE! clinics than veterans in the preperiod. Visit rates were higher among female veterans than male veterans (1.12; 95% CI, 1.10–1.15), among married veterans than unmarried veterans (1.04; 95% CI, 1.02–1.05), and among white veterans than nonwhite veterans (1.13; 95% CI, 1.11–1.15).

**Table 3:** Adjusted Model Estimates of Number of MOVE! Clinic Visits by New Users (N = 47,713)^a^

Variable	Estimate (95% CI)	*P* Value
Newly exempt	0.97 (0.94–1.00)	.06
Postperiod	0.98 (0.96–0.99)	.02
Newly exempt × postperiod interaction	1.00 (0.96–1.04)	.83
Age	1.005 (1.004–1.006)	<.001
Female	1.12 (1.10–1.15)	<.001
Married	1.04 (1.02–1.05)	<.001
White race	1.13 (1.11–1.15)	<.001
Comorbidity risk via diagnostic cost group score	1.01 (1.00–1.02)	.16
MOVE! clinic coordinated by primary care	0.93 (0.90–0.97)	.001
MOVE! coordinating committee includes . . .		
Nonprimary care nurse	1.09 (1.04–1.13)	<.001
Primary care physician	1.15 (1.11–1.20)	<.001
Midlevel provider	0.98 (0.94–1.02)	.36
MOVE! clinic has sufficient space to meet needs	1.25 (1.20–1.31)	<.001
MOVE! indoor exercise facility is sufficient to meet needs	0.75 (0.71–0.79)	<.001

Abbreviation: CI, confidence interval.

a Estimates, 95% CIs, and *P* values derived from a negative binomial mixed-effects model.

## Discussion

We examined the effect of eliminating the $15 MOVE! clinic copayment on veterans’ demand for MOVE! services and found a modest but not significant increase in MOVE! clinic use by veterans newly exempt from the MOVE! clinic copayment. The lack of significant increased use by newly exempt veterans is surprising; prior studies have shown copayment elimination to be associated with increased medication adherence (11) and financial incentives to be associated with initiation of exercise and weight loss (17–19). Several studies have also shown health club vouchers or direct payments to be associated with increased overall physical activity level (18–20), particularly when coupled with a series of motivational interviews (21). Financial incentives in a structured work health program increased exercise levels in high-risk patients but had little response among lower-risk patients (22). The pool of newly exempt veterans wanting to attend MOVE! clinic visits may have been limited, so the clinic-standardized rate of 5.8% by these veterans satisfied this previously unmet demand.

The increase in MOVE! clinic use in the year after copayment elimination among both exempt and nonexempt veterans may have resulted from the continued efforts to promote the MOVE! program since 2006, rather than a direct result of the copayment elimination. The introduction of an obesity screening quality indicator in the latter half of 2008 (when the postperiod started) may have increased demand for MOVE! services by both patients and VHA clinicians. The increase in MOVE! use by exempt veterans after copayment elimination suggests that VHA does not need to be concerned about “a run on services” following copayment elimination. Exempt veterans may have used MOVE! at a higher rate than newly exempt veterans because they have greater VA use in general. To increase MOVE! use among newly exempt veterans, VA may want to consider direct financial incentives to veterans instead of a copayment reduction. A copayment reduction may be less motivating to individuals compared with a deposit contract approach (23), direct payments for weight reduction (19,24), or discounting of fitness services (25). Provision of financial incentives may be more effective for initiation of MOVE! visits than for intense and sustained MOVE! use (18,26), and incentives also might be more effective if combined with provider-level interventions.

This study has several limitations. First, these results may not generalize to MOVE! clinics that had low volume in the year before the copayment elimination or to more recent years (ie, mid-2009 and after). Second, we do not have baseline obesity and overweight status for the exempt and nonexempt groups. Higher-income, nonexempt veterans may have had fewer candidates for participation in the MOVE! program, which could account for the lower enrollment increase in that population. Third, we did not have data on the number of veterans who wanted to participate in the MOVE! program but were unable to participate. If a disproportionate share of veterans required to pay copayments were placed on waiting lists to enroll in MOVE!, these results would underestimate the response to the copayment elimination by these veterans because they only reflect actual MOVE! clinic visits. Fourth, there may have been changes in MOVE! clinics themselves over time that created maturation biases that potentially confounded the effect of the copayment elimination. Fifth, the examination of the MOVE! clinic copayment using a cross-sectional time-series study design of new MOVE! clinic users, some of whom were newly exempt veterans and some of whom were nonequivalent “always exempt” veterans, may have limited the internal validity of this evaluation. However, examination of MOVE! clinic demand by new users and inclusion of a control group (albeit nonequivalent) controlled for regression-to-the-mean bias and other threats to internal validity.

Eliminating the copayment reduced a financial barrier to access the MOVE! clinic, yet we did not observe increased use by veterans newly exempt from these copayments compared with veterans who were already exempt from copayments. Unexpectedly, we saw a larger increase in demand among veterans who receive all VA care for free, which may have been driven by clinic staff perception of greater potential benefit of MOVE! clinic use in this segment of the population or by a greater perceived value of this exemption. Copayment elimination may be less important for health behavior change services that require ongoing commitment from patients. These results suggest that VA should not assume that copayment reductions for selective preventive services will motivate patient change and achieve intended system-level outcomes.
